# Comparative Study on the Outcomes of Elective-Start versus Urgent-Start Peritoneal Dialysis Catheter Placement

**DOI:** 10.1155/2020/3751827

**Published:** 2020-04-25

**Authors:** Ahmed Kamel Abdel Aal, Khalid Mahmoud, Amr Soliman Moustafa, Noha Alaaeldin Aboueldahab, Anas Souid, Andrew Gunn, Yufeng Li, Zhixin Wang, Ammar Almehmi

**Affiliations:** ^1^Department of Diagnostic and Interventional Imaging, University of Texas Health Science Center at Houston, Houston, TX 77030, USA; ^2^Department of Radiology, University of Alabama at Birmingham, Birmingham, AL 35233, USA; ^3^Department of Radiology, University of Arkansas for Medical Sciences, Little Rock, AR 72205, USA; ^4^Department of Radiology, Zagazig University, Zagazig, Egypt; ^5^Department of Medicine, Division of Preventive Medicine, University of Alabama at Birmingham, Birmingham, AL 35233, USA; ^6^Department of Medicine, Division of Nephrology, University of Alabama at Birmingham, Birmingham, AL 35233, USA

## Abstract

The aim of this study is to compare the outcomes of the elective-start versus urgent-start use of peritoneal dialysis (PD) catheters using percutaneous radiologic or laparoscopic techniques. Patients having their first peritoneal dialysis catheter placed and used between January 2005 and January 2018 were identified, and their medical records were retrospectively reviewed. Two groups were identified: elective-start (*n* = 211) and urgent-start (*n* = 29). Patient's demographics were similar between the two groups with the exception of age, which was higher in the elective-start group. The catheter complication rates and catheter removal rates at 3 and 12 months, mean days-to-first complication, mean days-to-catheter removal, and overall patient survival at 12 months were analyzed. Catheter complication rates at 3 and 12 months were similar between the two groups (27.8% and 48.9%, respectively, in the elective-start group versus 35.9% and 54.2%, respectively, in the urgent-start group, *p*=0.415). The catheter removal rates at 3 and 12 months were also similar between the two groups (*p*=0.088). Catheter leak was higher in the urgent-start group (13.8% versus 3.3%, respectively, *p*=0.011). There was no difference between the elective-start and the urgent-start groups in the mean days-to-first complication (95 vs 69, *p*=0.086), mean days-to-catheter removal (145 vs 127, *p*=0.757), and overall patient survival at 12 months (100% vs 97%, *p*=0.41). In conclusion, apart from catheter leak, there were similar rates of catheter complication and removal for PD catheter used for the elective-start compared to the urgent-start PD. Furthermore, the technique of placement did not affect the outcomes.

## 1. Introduction

Chronic kidney disease (CKD) as well as end-stage renal disease (ESRD) is prevalent throughout the world with an incidence of 13.6% and 0.14%, respectively [1]. More than 25,000 patients in the United States receive dialysis via peritoneal dialysis (PD) [2]. PD is a patient-centered cost-effective modality compared to hemodialysis (HD) [3]. This is because PD can be carried out at home, affording unique lifestyle benefits for patients, and allowing for lower dialysis costs [4]. PD also provides preservation of kidney function [5] and improved mortality [6] when compared to HD.

It is expected that PD will be utilized at a higher rate in the United States as recent modifications by the Centers for Medicare and Medicaid Services (CMS) resulted in higher reimbursement for PD services [7]. The distinct advantages of PD have led to the “PD First” position by some clinicians and to consider PD for urgent dialysis commencement and not only for the elective-start dialysis settings [8–10]. The urgent-start dialysis is a term used to describe patients needing immediate dialysis initiation with no preestablished vascular or PD access, where initiation of the PD occurs within 2 weeks after catheter insertion [8]. Therefore, the urgent-start PD is an attractive option for patients presenting with ESRD without prior access preparation [11].

Nevertheless, PD in the urgent-start setting is frequently underutilized for several reasons. First, patients may have difficulty making a decision regarding a dialysis modality. Second, there may be limited experience with PD or a lack of resources at multiple levels, leading to reluctance in using PD especially in patients with severe uremic symptoms and volume overload. Third, the urgent-start PD requires prompt PD catheter placement (typically within 24 to 48 hours of presentation), which might not be available in all institutions and medical facilities [12, 13]. While PD catheters are better in terms of overall morbidity and mortality, they are not entirely without risk. Abdominal wall complications, most commonly hernia and leak, may occur owing to the increased intra-abdominal pressure from high dialysate volume infused in the peritoneal cavity [14]. Technical failure from malpositioned catheters may also occur [15]. Data regarding the outcomes of the elective-start compared to the urgent-start PD is limited, with few published studies available in the literature [16–18].

The purpose of this study was to compare the outcomes of PD catheter placement in the elective-start versus urgent-start settings.

## 2. Methods

### 2.1. Study Population

This study was approved by the local institutional review board, and informed consent was waived. A total of two hundred and sixty patients who underwent PD catheter placement between January 2005 and January 2018 were retrospectively reviewed. Two groups were identified: the elective-start group, which comprised of patients who had PD catheters inserted as part of patient preparation for dialysis access, and, the urgent-start group, which included patients who presented with no preestablished access and had their PD catheters placed for the immediate initiation of dialysis. PD catheter insertion was performed either by an interventional radiologists and nephrologists using percutaneous, image-guided technique (fluoroscopy and ultrasound guidance) using conscious sedation, or by surgeons using laparoscopic technique using general anesthesia. The decision to perform percutaneous image-guided versus laparoscopic placement of the catheters was mainly based on physician discretion taking into consideration the age and comorbidity of the patients, and the ability to perform general anesthesia. Older patients with comorbid conditions who were not eligible to obtain general anesthesia were referred for percutaneous catheter placement.

Some of the study subjects may overlap with another study titled “Outcomes of fluoroscopic and ultrasound-guided placement versus laparoscopic placement of peritoneal dialysis catheters,” which was published in the Clinical Kidney Journal [19]. The objective of that study was to compare the fluoroscopy and ultrasound guidance technique with the laparoscopic technique. This study focuses on comparing the outcomes of PD catheter placement in the elective-start versus urgent-start settings regardless of the technique.

**Figure 1:**
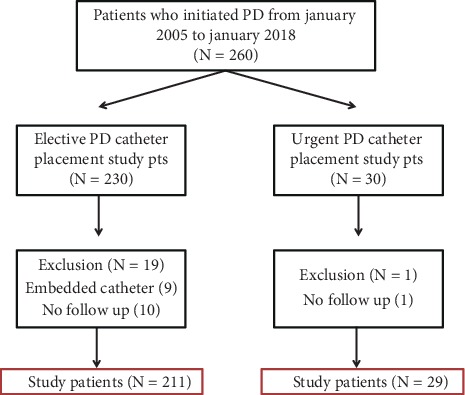
Algorithm for patients' inclusion and exclusion.

Patients with stage-5 CKD or with ESRD, age ≥19 years, and with PD catheter placed during the study period were the inclusion criteria. Exclusion criteria were patients with no documentation of follow-up at our institution or those with a PD catheter buried under the skin and not used during the study period due to the patients who did not meet the criteria for dialysis (embedded catheters). Patients who underwent laparoscopic adhesiolysis, hernia repair, or omentopexy, patients who had previous abdominal surgery, or patients with severe obesity, defined as BMI of 30 or higher, were included in both groups.  Figure 1 illustrates the algorithm of including and excluding patients. Two hundred and forty patients were included in the study.

The demographics and comorbidities of the patients were obtained from the medical records. BMI was classified into 3 categories: Class-1 is BMI of 30 to <35, Class-2 is BMI of 35 to <40, and class-3 is BMI of 40 or higher. We also recorded the prior surgeries of the patients.

There were 211 patients in the elective-start group as well as 29 patients in the urgent-start group. Patients in both groups had similar demographics and medical comorbidities except for age, which was higher in the elective-start group (55.8 years, SD = 15.6) compared to the urgent-start group (47.2 years, SD = 15.9) (*p*=0.0094) (Table 1). The elective-start group consisted of 121 (57.3%) females and 90 (42.7%) males, while the urgent-start group included 16 (55.2%) males and 13 (44.8%) females. Obesity was similar in the elective-start and urgent-start groups and was seen in 73 patients (34.6%) and 12 patients (41.3%), respectively (*p*=0.47). The mean BMI was also similar between the elective-start group (29.2) and the urgent-start group (28.4) (*p*=0.855). In the elective-start group, 32 patients (15.2%), 26 patients (12.3%), and 15 (7.1 %) were class-1, class-2, and class-3 obesity, respectively. In the urgent-start group, 8 patients (27.6%), 3 patients (10.3%), and 1 patient (3.4%) were class-1, class-2, and class-3 obesity, respectively. Prior surgical procedures were performed more commonly in the elective-start group (117 patients, 55.5%) compared to the urgent-start group (10 patients, 34.5%) (*p*=0.034) (Table 2).

### 2.2. Study Outcomes

Following PD catheter placement, the patients were followed up regularly by their nephrologists. Information on catheter complication and removal was obtained from the nephrology clinic notes in the patients' medical records.

The occurrence of PD catheter-related complications at 3 and 12 months was a composite endpoint that included infectious, mechanical, and technical complications. The infectious complications included the following: exit-site infection and tunnel infection as well as peritonitis. Peritonitis was defined as an infectious process that starts at the exit site, migrates along the subcutaneous tunnel, and ends in peritonitis. The mechanical complications included the following: catheter leakage through the exit-site, catheter malfunction caused by insufficient catheter drainage, and the development of abdominal hernia. The technical complications included the following: bowel perforation, muscle hematoma, intraperitoneal bleeding, and insertion failure resulting from either failing to insert the catheter or inability to use it after successful insertion. Comparison between radiologic and laparoscopic techniques in each of the two groups was performed.

Catheter removal at 3 and 12 months (catheter removal), mean days-to-first complication, mean days-to-catheter removal, and overall patient survival were calculated. All information regarding the catheter's placement, complications, and removal were acquired from the patients' electronic medical records.

### 2.3. Radiologic and Laparoscopic Technique of PD Catheter Placement

Percutaneous PD catheter insertion utilizing image-guided technique (fluoroscopy and ultrasound) has been described in the literature [4, 20, 21]. The laparoscopic technique has likewise been described in the literature [22]. In the urgent-start group, dialysis was started within 48 hours of catheter insertion with low-volume dialysis until wound healing occurred. In the elective-start group, dialysis was started with large volume after complete wound healing, which typically occurs within 21 days.

### 2.4. Statistical Analysis

Descriptive analysis was presented for patient demographics, clinical history, imaging, and laboratory data, including mean, standard deviation, count, and percentage. To compare continuous variables between the two groups, two sample *t*-tests were conducted. Careful attention was given to the normality assumption, which was examined by normal probability plots and histograms. To compare categorical variables, Fisher's exact test was conducted. Time to complication and time to catheter removal were analyzed using the Kaplan–Meier (KM) method. The 3 and 12 months complication-free and catheter removal rates were estimated from KM curves. Time to complication and time to catheter removal were calculated from the date of procedure to the date of complication and the date of catheter removed within 12 months. Right censor was considered if patients lost at the end of the study or if the event did not occur within the study duration. For all inferences, the significant level was set to *p* ≤ 0.05. All analyses were conducted using SAS v.9.4 (SAS Inc., USA).

## 3. Results

Catheter complication rates are shown in Table 3. In both groups, the most common complications were catheter malfunction and peritonitis accounting for 28.4% and 15.1%, respectively, in the elective-start group compared to 17.2% and 27.6%, respectively, in the urgent-start group but without a significant difference (*p*=0.203 and *p*=0.092, respectively). Catheter leakage was more frequent in the urgent-start group when compared to the elective-start group (13.8% versus 3.3%, respectively, *p*=0.011). Bowel injury resulting in perforation was documented in one of the patients in the elective-start group and was placed using the image-guided technique. This patient was treated conservatively using prophylactic antibiotic administration.

**Figure 2:**
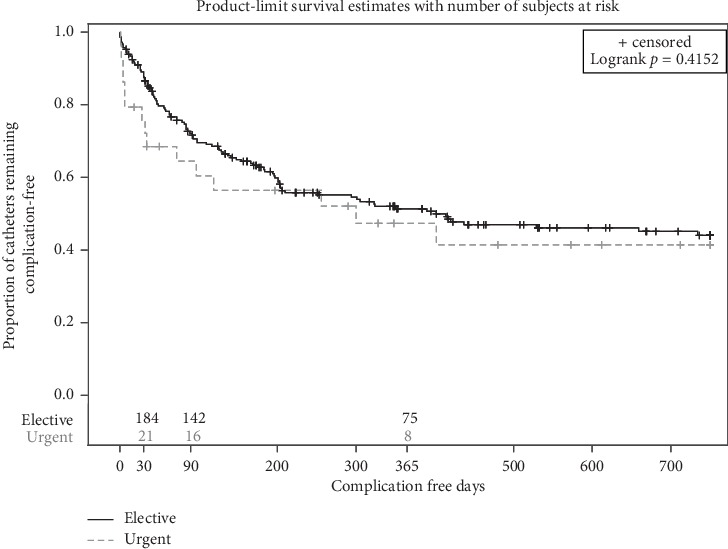
Kaplan–Meier curves for catheter complication rates in the elective-start and urgent-start groups, showing no difference between both groups.

**Figure 3:**
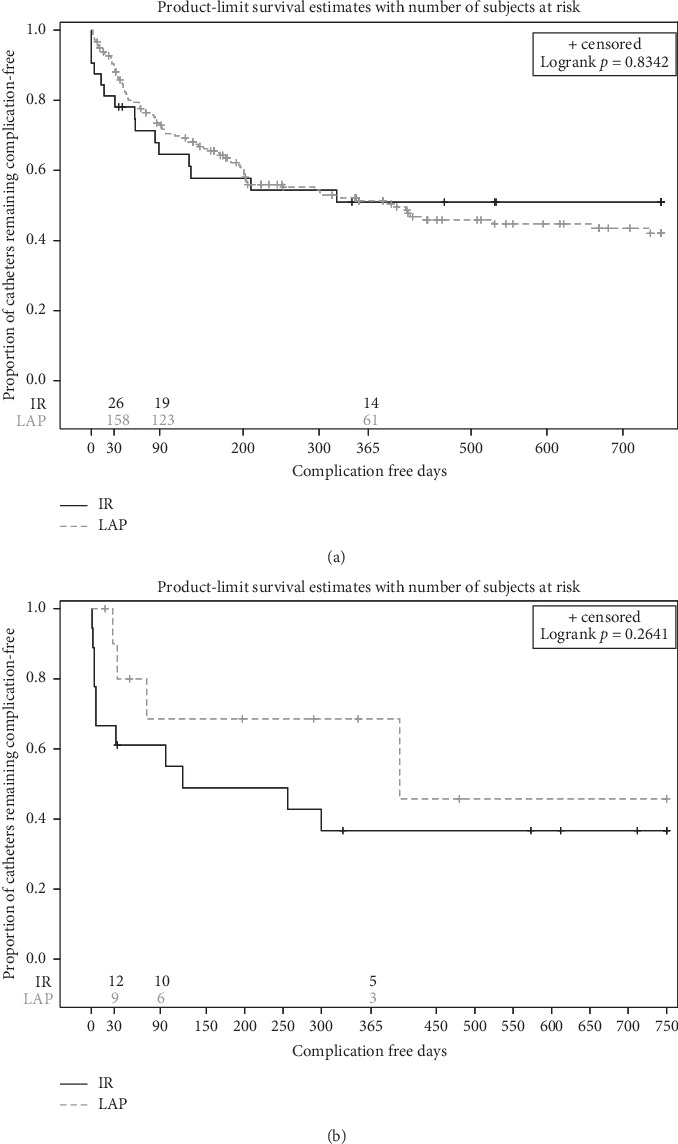
Kaplan–Meier curves for catheter complication rates based on radiologic and laparoscopic techniques in the (a) elective-start group and (b) urgent-start group. There was no difference in the complication rates based on the technique used for catheter placement.

Kaplan–Meier analysis revealed nonsignificant difference in the rates of catheter complications when comparing the two groups (*p*=0.415) (Figure 2). The estimated catheter complication rates at 3 and 12 months from the KM analysis were 27.8% and 48.9%, respectively, in the elective-start group versus 35.9% and 54.2%, respectively, in the urgent-start group (Table 4). When patients were compared based on the technique of catheter placement (radiologic versus laparoscopic) in each group separately, there was no difference in the rate of catheter complications between the two techniques (*p*=0.834 and *p*=0.264 for the elective-start and urgent-start groups, respectively) (Figures 3(a) and 3(b)). No difference was noted in the mean days-to-first complication which was 95 days (SD = 88, range 0–350 days) in the elective-start group compared to 69 days (SD = 97, range 1–300 days) in the urgent-start group (*p*=0.086).

**Figure 4:**
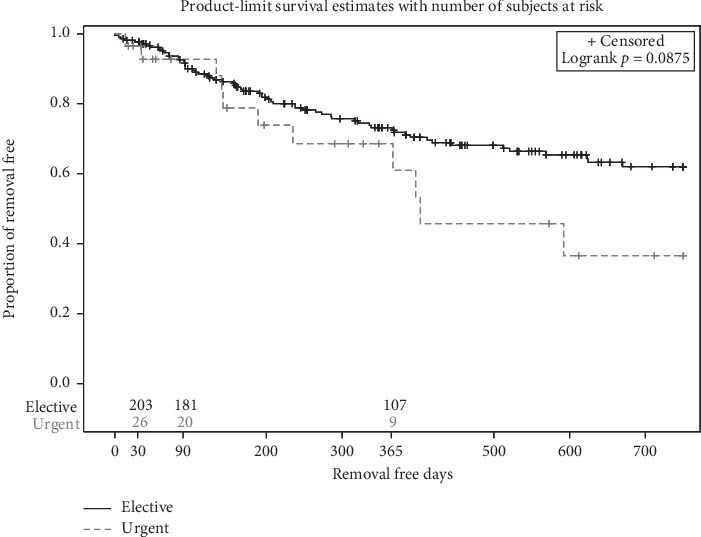
Kaplan–Meier curves for overall catheter removal in the elective-start and urgent-start groups showing no difference between the two groups.

Kaplan–Meier analysis demonstrated no difference in the overall catheter removal rates when comparing the two groups (*p*=0.088) (Figure 4). The estimated catheter removal rates from the KM analysis are shown in Table 4. The overall catheter removal at 3 and 12 months were 7.3% and 26.9%, respectively, in the elective-start group versus 7.7% and 34.8%, respectively, in the urgent-start group. There was no difference in the mean days-to-catheter removal, which was 145 days (SD = 95, range 0–338 days) in the elective-start group compared to 127 days (SD = 79, range 14–235 days) in the urgent-start group (*p*=0.757).

**Figure 5:**
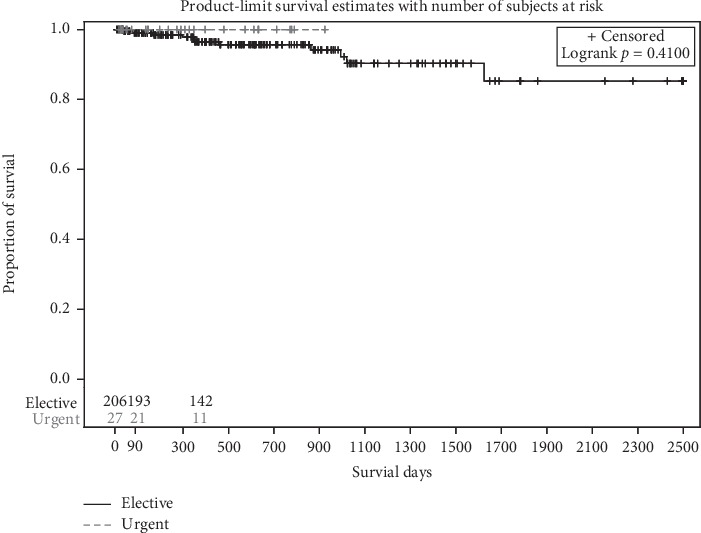
Kaplan–Meier curves for overall patient survival in the elective-start and urgent-start groups showing no difference in survival at 12 months.

Kaplan–Meier survival analysis revealed no difference in the overall survival of the patients when comparing the two groups (*p*=0.41) (Figure 5), although the mean age was higher in the elective-start group. The urgent-start group had no estimated deaths at 3 and 12 months compared to 1.0% and 3.33%, respectively, in the elective-start group. Four patients died in the first year in the elective-start group, and the causes of death were not related to the procedure or catheter complications, but were due to septic shock from pneumonia in one patient, multiorgan failure in one patient, and cardiopulmonary arrest in two patients.

## 4. Discussion

PD has several advantages over HD including an improved lifestyle for patients and enhanced outcomes [23, 24]. Survival superiority of PD over HD in the first two-years of therapy has been shown, with similar outcomes up to five years [25–29]. Typically, the late-referred patients who are good candidates for PD are usually started on HD using a temporary central venous catheter due to the inability to place PD catheter in a timely manner [16]. However, temporary central venous catheters are usually associated with increased risk of complications including but not limited to sepsis and bacteremia requiring hospitalization and catheter malfunction requiring catheter replacement [30].

PD catheter placement utilizing image-guided percutaneous methods is essential for rapid commencement of PD in late-presenting patients [31]. Various centers have established pathways for surgical and image-guided percutaneous PD catheter placements to provide rapid initiation of PD, in which assisted PD treatments are provided by dedicated PD nursing staff in the outpatient setting until clinical improvement, and then, the patient is trained on self-care at home [31–33].

Rapid placement of PD catheters with rapid initiation of dialysis allows for a single procedure to provide both short-term and long-term access. However, these patients frequently find themselves faced with the risk of dialysate leak by earlier use of the PD catheter [9, 16]. In the urgent-start setting, catheter leak is one of the most commonly encountered complications ranging 0–33.3% [9, 33–39]. Our study is consistent with the published literature showing a similar risk of leak with a rate of 13.8% for the urgent-start group, which was higher than that of the elective-start group. Several maneuvers have been described to minimize the risk of dialysate leak associated with PD catheter placement in the urgent-start setting. These include recumbent dialysis only with lower dwell volumes and avoidance of dialysate dwells while the patient is upright [9]. In the elective-start setting, patients typically initiate PD after 2–3 weeks to allow for tissue ingrowth of the deeper Dacron cuff, which significantly minimize the risk of dialysate leak into the subcutaneous tissue [9].

The complication-free catheter survival or catheter removal rates in each group did not differ based on the technique of PD catheter placement used. Nayak et al. [16] conducted a prospective case-controlled study on 56 patients comparing the outcomes of patients who underwent conventional PD fourteen days after catheter insertion with those who underwent the urgent-start automated PD within 48 hours of catheter placement. Exit-site leak, catheter blockage, and peritonitis at 90 days were similar in both groups, and the study concluded that urgent-start automated PD in the unplanned patient is an appropriate approach. Another recent study evaluated the safest and shortest interval to commence PD after catheter insertion [17]. Patients were randomized to 3 groups in which PD was started at 1 week, 2 weeks, and 4 weeks. Catheter leak was significantly higher in patients who initiated PD at 1 and 2 weeks compared to 4 weeks. The usage of manual exchanges in that study could have been responsible for the increased incidence of leaks.

A single-center study assessed 30 patients who started on urgent PD, which was initiated within 2 weeks of catheter insertion (range: 0–13 days, median = 6 days), with 66.7% of the PD catheters inserted percutaneously versus 33.3% inserted laparoscopically. Minor pericatheter leak developed in 10% of the patients during the first week of training was treated conservatively. Catheter migration resulting in dysfunction was seen in 20% of patients and was treated by repositioning, without any need for catheter exchange or change in dialysis modality. The study concluded that urgent-start PD is safe and a reasonable alternative option to hemodialysis for patients who need to start PD urgently with no preexisting dialysis access [34]. Several other retrospective studies supported the concept that shorter break-in periods are not associated with more catheter-related complications [9, 35, 36].

Comparison between radiologic and laparoscopic techniques for placement of peritoneal dialysis catheters has been previously published in the literature [19], with no significant difference in the safety and outcomes between the two techniques. This study adds to the evolving literature signifying that PD catheter placement in the urgent-start setting, regardless of the technique used for catheter placement whether radiologic or laparoscopic, is not inferior compared to placement in the elective-start settings and therefore may offer late-referred ESRD patients an alternative patient-centered dialysis option, while avoiding the side effects of placing a CVC.

Future directions will probably focus on continued improvement in the insertion techniques to prevent leaks, implementing protocols that address the urgent-start PD needs to facilitate patient transition to outpatient home dialysis training, and increasing the awareness of the urgent-start PD program to combat the deficits seen among the ER physicians, nephrologists, hospitalists, nursing staff, and discharge planners. The success of an urgent-start program depends on the institution of a multidisciplinary approach to ensure rapid initiation and proper monitoring of PD therapy and smooth transition to long-term PD therapy. This requires teamwork of dedicated interventional radiologists and surgeons, PD nurses, renal nutritionists, and the ancillary hospital resource staff including pharmacy [16].

Limitations of this current study include its retrospective design with selection-bias being an inherent disadvantage. In addition, the study describes a single-center experience, and the results cannot be generalized. Clinical outcomes such as duration of hospital stay and mortality were not included in the analysis.

In conclusion, apart from the higher incidence of catheter leak, the outcomes of PD catheters in the urgent-start setting are not inferior to the elective-start setting with similar complication-free catheter survival and catheter removal rates at 3 and 12 months regardless of the technique used for catheter placement. These findings suggest that urgent-start PD is as safe as elective-start PD and has the advantage of avoiding the side effects and complications associated with CVC placement in late-referred ESRD patients who are considered for PD.

## Figures and Tables

**Table 1:** Demographics and comorbidities of the elective-start and urgent-start groups.

Characteristics	Elective-start group, *N* = 211 (%)	Urgent-start group, *N* = 29 (%)	*p* value
Age^*∗*^	55.8 (15.6)	47.2 (15.9)	0.009
Sex			0.203
Female	121 (57.3)	13 (44.8)	
Male	90 (42.7)	16 (55.2)	
Body Mass index (BMI)^*∗*^	29.2 (7.4)	28.4 (6.8)	0.855
Diabetes	100 (47.4)	15 (51.8)	0.662
Hypertension	193 (91.5)	29 (100)	0.102
Coronary artery disease	53 (25.1)	6 (20.7)	0.604
Congestive heart failure	52 (24.6)	5 (17.2)	0.380
Peripheral vascular disease	26 (12.3)	3 (10.3)	0.759
Cerebrovascular disease	13 (6.2)	1 (3.5)	0.559

^*∗*^Mean (Standard deviation).

**Table 2:** Prior surgical procedures in the elective-start and urgent-start groups.

Type of surgery	Elective-start group, *N* = 211 (%)	Urgent-start group, *N* = 29 (%)	*p* value
Total no. of surgeries	117 (55.5)	10 (34.5)	0.034
Abdominal exploration	4 (1.9)	0 (0)	
Appendectomy	10 (4.7)	1 (3.4)	
Caesarean section	26 (12.3)	2 (6.9)	
Cholecystectomy	29 (13.7)	0 (0)	
Colon surgery	3 (1.4)	0 (0)	
Fundoplication	1 (0.5)	0 (0)	
Gastric bypass	4 (1.9)	0 (0)	
Hernia repair	21 (10)	2 (6.9)	
Hysterectomy	40 (19)	1 (3.4)	
Kidney transplantation	18 (8.5)	3 (10.3)	
Myomectomy	1 (0.5)	0 (0)	
Nephrectomy	13 (6.2)	1 (3.4)	
Salpingo-oophorectomy	7 (3.3)	1 (3.4)	
Tubal ligation	13 (6.2)	0 (0)	

**Table 3:** Catheter complication rates for the elective-start and urgent-start groups.

	Elective-start group, *N* = 211 (%)	Urgent-start group, *N* = 29 (%)	*p* value
Exit site infections	11 (5.2)	1 (3.5)	0.683
Peritonitis	32 (15.1)	8 (27.6)	0.092
Catheter malfunction	60 (28.4)	5 (17.2)	0.203
Catheter leak	7 (3.3)	4 (13.8)	0.011
Primary leak	2 (1)	1 (3.5)	0.256
Hernia	9 (4.3)	3 (10.3)	0.159
Muscle hematoma or bleeding	6 (2.8)	0 (0)	0.358
Bowel perforation	1 (0.5)	0 (0)	0.710

**Table 4:** Estimated catheter complications and removal rates for the elective-start and urgent-start groups from the Kaplan–Meier curves.

	Elective-start group, *N* = 211 (%)	Urgent-start group, *N* = 29 (%)	*p* value^*∗*^
Catheter complication rate at 3 months	27.8	35.9	0.415
Catheter complication rate at 12 months	48.9	54.2	
Catheter removal at 3 months	7.3	7.7	0.088
Catheter removal at 12 months	26.9	34.8	

^*∗*^Logrank test.

## Data Availability

The data used to support the findings of this study are available from the corresponding author upon request.

## Abbreviations and acronyms

CKD: Chronic kidney disease
ESRD: End-stage renal disease
PD: Peritoneal dialysis
HD: Hemodialysis
CMS: Centers for Medicare and Medicaid Services
KM: Kaplan–Meier.
